# Oxidative Stress and New Pathogenetic Mechanisms in Endothelial Dysfunction: Potential Diagnostic Biomarkers and Therapeutic Targets

**DOI:** 10.3390/jcm9061995

**Published:** 2020-06-25

**Authors:** Maria Giovanna Scioli, Gabriele Storti, Federico D’Amico, Roger Rodríguez Guzmán, Federica Centofanti, Elena Doldo, Ela María Céspedes Miranda, Augusto Orlandi

**Affiliations:** 1 Department of Biomedicine and Prevention, Anatomic Pathology Institute, Tor Vergata University of Rome, 00133 Rome, Italy; scioli@med.uniroma2.it (M.G.S.); fededamico92@gmail.com (F.D.); federica.centofanti@uniroma2.it (F.C.); elena.doldo@uniroma2.it (E.D.); 2 Department of Surgical Sciences, Plastic and Reconstructive Surgery, Tor Vergata University of Rome, 00133 Rome, Italy; gabriele.storti@uniroma2.it; 3 Biomedical Sciences Department, Calixto García Faculty, University of Medical Sciences of Havana, Havana 11600, Cuba; doctorhabana@gmail.com (R.R.G.); elaces@infomed.sld.cu (E.M.C.M.)

**Keywords:** cardiovascular diseases, endothelial dysfunction, oxidative stress, biomarkers, therapeutic targets

## Abstract

Cardiovascular diseases (CVD), including heart and pathological circulatory conditions, are the world’s leading cause of mortality and morbidity. Endothelial dysfunction involved in CVD pathogenesis is a trigger, or consequence, of oxidative stress and inflammation. Endothelial dysfunction is defined as a diminished production/availability of nitric oxide, with or without an imbalance between endothelium-derived contracting, and relaxing factors associated with a pro-inflammatory and prothrombotic status. Endothelial dysfunction-induced phenotypic changes include up-regulated expression of adhesion molecules and increased chemokine secretion, leukocyte adherence, cell permeability, low-density lipoprotein oxidation, platelet activation, and vascular smooth muscle cell proliferation and migration. Inflammation-induced oxidative stress results in an increased accumulation of reactive oxygen species (ROS), mainly derived from mitochondria. Excessive ROS production causes oxidation of macromolecules inducing cell apoptosis mediated by cytochrome-c release. Oxidation of mitochondrial cardiolipin loosens cytochrome-c binding, thus, favoring its cytosolic release and activation of the apoptotic cascade. Oxidative stress increases vascular permeability, promotes leukocyte adhesion, and induces alterations in endothelial signal transduction and redox-regulated transcription factors. Identification of new endothelial dysfunction-related oxidative stress markers represents a research goal for better prevention and therapy of CVD. New-generation therapeutic approaches based on carriers, gene therapy, cardiolipin stabilizer, and enzyme inhibitors have proved useful in clinical practice to counteract endothelial dysfunction. Experimental studies are in continuous development to discover new personalized treatments. Gene regulatory mechanisms, implicated in endothelial dysfunction, represent potential new targets for developing drugs able to prevent and counteract CVD-related endothelial dysfunction. Nevertheless, many challenges remain to overcome before these technologies and personalized therapeutic strategies can be used in CVD management.

## 1. Introduction

Cardiovascular disease (CVD) is a class of conditions affecting the heart and blood vessels, and represents the leading cause of morbidity and mortality in the world [1,2]. Oxidative stress and endothelial dysfunction play a central role in the pathogenesis of several vascular and metabolic human diseases, such as peripheral vascular disease, stroke, heart disease; and diabetes [3]. Endothelial activation is a pro-inflammatory and pro-coagulant state characterized by the expression of endothelial cell-surface adhesion molecules that are required for inflammatory cell recruitment; it is induced by cytokines produced by tissues under inflammatory conditions [3]. Endothelial function is influenced by several pathophysiological conditions; including hyperglycemia, hyperlipidemia, hypertension, as well as aging and exposure to specific drugs that can influence molecular mechanisms regulating nitric oxide (NO) bioavailability [4]. Endothelial dysfunction has been defined as a systemic insidious and reversible pathological state of the endothelium, derived from a reduced NO bioavailability and impaired vasodilation associated with pro-inflammatory and prothrombotic status [5,6,7,8]. Increased oxidative stress has been linked to impaired endothelial function and may play a role in the pathogenesis of the adverse effects of CVD [9]. The most relevant oxidative stress mechanisms involved in cardiovascular diseases are summarized in Table 1. 

**Figure 1:**
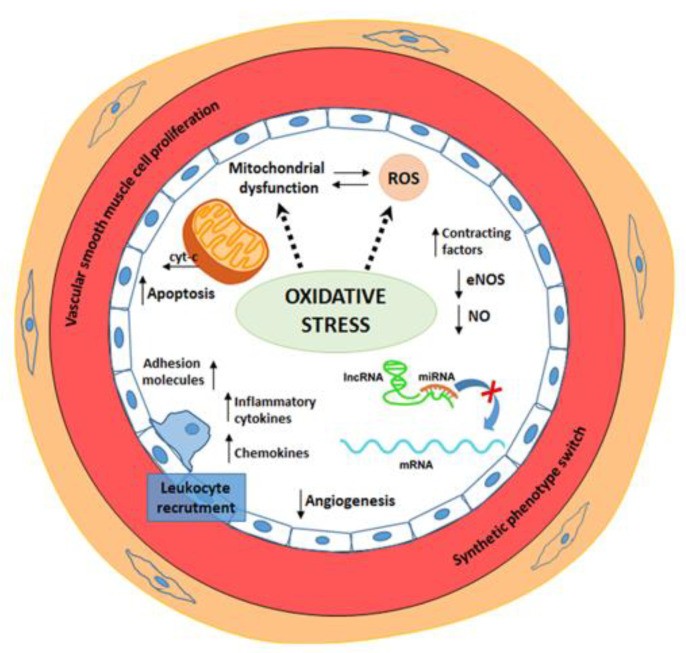
Schematic representation of the main pathogenetic mechanisms involved in endothelial dysfunction. Endothelial dysfunction is defined as an impaired vasodilation and a proinflammatory and prothrombotic status. Endothelial dysfunction-induced phenotypic changes include eNOS inactivation and diminished availability of NO, up-regulated expression of adhesion molecules and increased chemokine secretion, leukocyte adherence, cell permeability, low-density lipoprotein oxidation, platelet activation, and vascular smooth muscle cell proliferation and migration. Inflammation-induced oxidative stress results in an increased accumulation of reactive oxygen species (ROS), mainly derived from mitochondria. Excessive ROS production causes oxidation of macromolecules inducing cell apoptosis mediated by cytochrome-c release (cyt-c). Oxidative stress increases vascular permeability, promotes leukocyte adhesion and induces alterations in endothelial signal transduction and redox-regulated transcription factors. Recently, alterations of gene regulatory mechanisms and deregulation of non-coding RNAs (miRNAs, lncRNA) have been reported to contribute to endothelial dysfunction.

Mitochondria are an essential cellular structure in energetic metabolism and maintain a delicate regulatory balance between Ca^2+^ concentration and the production of NO [43]. Moreover, mitochondria are the primary intracellular source of reactive oxygen species (ROS), a toxic product of oxidative energy metabolism [44]. Excessive ROS production leads to oxidation of macromolecules and has been implicated in mtDNA mutations, aging, and cell apoptosis, induced by cytochrome-c release from its binding site to cardiolipin, which anchors it to the inner mitochondrial membrane [45]. Oxidation of cardiolipin reduces cytochrome-c binding and results in an increased level of “free” cytochrome-c in the intermembrane space [12]. Therefore; cardiolipin plays a vital role in mitochondrial-dependent steps of apoptosis; it is particularly susceptible to ROS attacks [46]. Another critical factor is the p66Shc adaptor protein that acts as a redox enzyme and is implicated in age-induced changes in endothelial function and hyperglycemia-induced endothelial dysfunction [47,48,49]. Different chronic stimuli can induce the phosphorylation of p66Shc, which translocates from the cytosol to the mitochondrion producing ROS; thus, determining the reduction of NO bioavailability, mitochondrial disruption, and cell apoptosis via cytochrome c releasing [48]. Oxidative-stress induced ROS generation contributes to mitochondrial damage, endothelial dysfunction, and promotes leukocyte adhesion, inflammation, thrombosis, and smooth muscle cell proliferation [44]. A schematic representation of the main pathogenetic mechanisms involved in endothelial dysfunction is reported in Figure 1.

Identifying new markers of oxidative stress represents a research goal for prevention, clinical diagnosis, and monitoring of endothelial dysfunction and adverse events of CVD [50]. Since CVD is a multifactorial disease, therapeutic strategies aim to improve insulin sensitivity, glycemic control, lipid profile, and blood pressure. The efficacy of these therapies often consists of endothelial dysfunction reversal [3]. As endothelial dysfunction can be reversible, the final goal of different treatments is to restore endothelium to its quiescent state [51]. In that sense, several pharmacological agents have been already tested in clinical settings: angiotensin-converting enzyme (ACE) inhibitors and angiotensin II type 1 (AT1) blockers, beta-blockers, Ca2+ channel blockers, phosphodiesterase-5 (PDE-5) inhibitors, statins, antiplatelet agents, statins, eNOS transcription enhancer, and renin-angiotensin-aldosterone system (RAAS) inhibitors [52]. The inhibitors of eNOS uncoupling represent another potential strategy to reduce ROS and have already been introduced into the clinical practice, such as the RAAS inhibitors, statins, metformin, and pentaerythritol tetranitrate [52]. New generation drugs based on Rho-associated protein kinase (ROCK) inhibitors, Poly (ADP-ribose) polymerase (PARP) inhibitors, protein tyrosine phosphatases (PTPs) inhibitors, geranylgeranyl transferase inhibitors, and transketolase, have proven effective against endothelial dysfunction [51,53,54,55,56]. Recently, gene regulatory mechanisms, such as histone acetylation/deacetylation, and changes in non-coding RNAs expression have been reported to contribute to vascular homeostasis, representing potential new targets for developing drugs able to prevent and counteract atherosclerosis [57,58]. Different experimental studies, based on carriers, gene therapy, enzyme inhibitors, have been carried out in order to discover new effective and personalized treatments [59,60,61,62,63]. Considering traditional modifiable risk factors is useful, but is not sufficient to obtain effective individual treatment or to predict response to therapy. The inclusion of relevant information derived from genetic and epigenetic modifications may help identify a more individualized functional risk profile in order to personalize treatment, monitor progression, and manage diseases. The final aim is to be able to identify at-risk patients who can be preventively treated with personalized therapies before they could develop symptoms; thus, reducing cardiovascular morbidity and mortality.

In this review, we discussed the role of new pathogenetic mechanisms in endothelial dysfunction, with particular attention to oxidative stress and gene regulatory networks. In light of recent findings, new biomarkers and the impact of novel personalized therapeutic approaches to endothelial dysfunction and CVD will be discussed.

## 2. Oxidative Stress and Endothelial Dysfunction and CVD

### 2.1. NADPH Oxidase (NOX) Activity and ROS Production

As previously reported, ROS plays a pivotal role in CVD, including hypertension, atherosclerosis, diabetes, cardiac hypertrophy, and heart failure. However, physiological ROS production is essential for the maintenance of normal vascular homeostasis [64]. In the vasculature, several enzyme systems, differentially localized and expressed, contribute to ROS formation. These include the nicotinamide adenine dinucleotide phosphate (NADPH) oxidase, endothelial nitric oxide (NO) synthase, enzymes of the respiratory chain, cytochrome P450 monooxygenase, and xanthine oxidase [64]. Among these systems, NADPH oxidase seems to play a central role in orchestrating the activation and dysfunction of other enzymes. It is the main source of ROS in the vessel wall [64,65]. In general, it is accepted that under physiologic conditions, vascular NADPH oxidases have a relatively low level of constitutive activity that exerts an important role in the cardiovascular system homeostasis [64,65,66]. However, enzyme activity can be increased both acutely and chronically in response to stimuli such as cytokines, growth factors, hyperlipidemia, and high glucose, which can impair vascular functions and cause CVD [64]. Different isoforms named Nox1, Nox2, Nox4, and Nox5 are expressed in the human vasculature. Nox4 is the predominant isoform in endothelial cells and mainly produces H_2_O_2_, which might explain the functional differences compared to other Nox isoforms producing superoxide anion (O2-) [67]. The specific contribution of each Nox isoform in cardiovascular physiology and disease remains unclear. Several studies on animal models have been performed to clarify the role of Nox isoforms in CVD. For example, Nox1 overexpression in the media has been reported in increased neo-intima formation after vascular injury [64]. Moreover, Nox1 and Nox5 are proven to induce O2- formation and favor human vascular smooth muscle cells (VSMC) proliferation [68]. Nox5 can contribute to the oxidative stress in human coronary artery disease [67]. The upregulation of Nox1 and Nox2 in vascular cells has been demonstrated to contribute to atherosclerosis and vascular diseases [67]. The association between endothelial dysfunction and Nox2 has been studied in several conditions such as dyslipidemia, obesity, smoking, hypertension, and aging [68]. Nox2 levels were found increased in the aortas of apolipoprotein E (ApoE)−/− atherosclerotic mice, whereas mice overexpressing Nox2 showed endothelial activation [64,68]. The relationship between Nox2 and aging has been investigated in ApoE−/− mice, which showed an upregulation of Nox2 in atherosclerotic plaque [68]. Endothelial-specific overexpression of Nox2 in transgenic mice has been reported to enhance angiotensin II-induced endothelial dysfunction contributing to vascular remodeling and hypertension [69]. However, in humans, the genetic deficiency of Nox2 is associated with enhanced endothelium-dependent flow-mediated vasorelaxation, decreased markers of vascular aging and oxidative stress [68]. In coronary arteries, patients with a congenital heart disease were found to have enhanced superoxide production in association with the upregulation of Nox2 [70]. Recent studies have put attention on the major physiologic and pathological roles of Nox4. Nox4 has been reported to contribute to oxidative stress, but evidence from Nox4−/− mice suggests that endogenous Nox4 has a vasoprotective function during ischemic or inflammatory stress [64]. Some animal studies showed that a genetic deficiency of Nox4 was associated with endothelial dysfunction and increased atherosclerosis burden [68]. However, Nox4−/− mice were protected from oxidative stress, blood–brain barrier leakage, and neuronal apoptosis in a stroke model [71]. In another model of cardiac-specific deletion of Nox4, cardiac hypertrophy, fibrosis, and apoptosis after pressure overload was reduced, suggesting different cell and dose-dependent responses of Nox4 in CVD [67]. It has also been postulated that increased activity of Nox4 or a putative “uncoupling” of Nox4 may switch its activity from H_2_O_2_ to O2- formation explaining its deleterious effects on vascular functions [65].

### 2.2. Antioxidant Enzymes in Endothelial Dysfunction

Antioxidants are molecules that can inhibit oxidation of other molecules, through scavenging oxidants or decreasing the production of ROS [72]. Based on their origins, small molecule antioxidants can be endogenous (e.g., uric acid, coenzyme Q, bilirubin) or exogenous (e.g., vitamins C and E, flavonoid, carotenes). Major antioxidant systems present in the vascular wall include superoxide dismutase, catalase, glutathione peroxidase, thioredoxin, peroxiredoxin [73]. The dysregulation of oxidant and antioxidant enzymes, such as an inefficient degradation of ROS, an increased activity of oxidant enzymes or a reduced/aberrant activity of antioxidant enzymes, produces an increase in ROS levels which overcome the buffering capacity of ROS scavengers causing oxidative stress [74].

#### 2.2.1. Superoxide Dismutase

Superoxide dismutase (SOD) belongs to a family of enzymes that catalyze the conversion of the O2- to H_2_O_2_ [3]. Three isoforms of SODs have been described: SOD1 is found in the cytoplasm and on the inner mitochondrial membrane; SOD2 is located in the mitochondrial matrix, and SOD3 is extracellular [73]. In endothelial cells, it has been reported that inhibition of SOD1 increased the steady-state levels of O2- and reduced the phosphorylation of extracellular signal-regulated kinases (ERK) 1/2. These inhibited extracellular angiogenetic signals are mediated by fibroblast growth factor-2 (FGF-2) and vascular endothelial growth factor (VEGF). Besides, increased superoxide levels and vascular relaxation impairment were found in the aorta of SOD2-deficient animals, in a model of atherosclerosis [3]. It has been reported that metabolic disorders characterized by excessive ROS production, such as diabetes, can alter the activity of SOD2, inducing endothelial dysfunction. In endothelial progenitor cells (EPCs) isolated from diabetic mice, reduced SOD2 levels associated with impaired wound healing and angiogenesis [75]. Moreover, reduced levels of SOD3 isoform were found to be correlated with increased ROS levels in the aorta of aged rats [76], whereas SOD3 overexpression has been shown to improve endothelial function in rat models of hypertension and heart failure [3].

#### 2.2.2. Glutathione Peroxidase

Glutathione peroxidase (GPx) is a selenium-containing antioxidant enzyme that reduces hydrogen peroxide and lipid peroxides into water and lipid alcohols, respectively, and in turn, oxidizes glutathione into glutathione disulfide (GSSG). In the absence of adequate GPx activity or glutathione levels, hydrogen peroxide and lipid peroxides are not detoxified and may be converted to hydroxyl radicals and lipid peroxyl radicals [74]. In humans, four isoforms of GPxs are known, each with the selenocysteine at the active site. The most abundant cellular form of GPx is GPx-1. GPx-1 deficiency has been linked to the development of endothelial dysfunction, inflammation, and neointimal formation [77]. The role of GPx-1 has been demonstrated in the normalization of VSMC proliferation rate through the inhibition of matrix metalloproteinase 9 (MMP9), and the consequent restoring of the endothelial function [73]. A more aggressive atherosclerosis has been reported in double knockout (ApoE−/−) (GPx-1−/−) mice compared to (ApoE−/−) controls, with a marked increase in ROS levels and a decrease in NO bioavailability, which was associated with increased protein nitration within the atherosclerotic lesions [78].

#### 2.2.3. Catalase

Catalase is a homotetrameric heme-containing protein located exclusively in the peroxisome and catalyzes hydrogen peroxide reaction into water and oxygen [3]. An overexpression of catalases and SOD-1 has demonstrated an atheroprotective effect with a decrease in the content of F2-isoprostanes in (ApoE−/−) mice aortas. Increased activity of catalases was found in foam cells derived from atherosclerotic lesions in rabbit aortas (Anastasia V Poznyak Biology (Basel) 2020). The enzyme is especially crucial when facing limited glutathione content or reduced GPx activity and plays a significant role in the development of tolerance to oxidative stress in the adaptive response of cells [74]. Catalase actively participates in the adaptive response of cells to oxidative stress and may be induced under oxidative factors, such as oxidized low-density lipoproteins (oxLDL), in endothelial cells [3].

#### 2.2.4. Thioredoxin

Thioredoxin system consists of both thioredoxin (Trx) and thioredoxin reductase (TrxR) that can reduce hydrogen peroxide and target proteins. The two mammalian isoforms Trx (Trx1 and Trx2) and three isoenzymes of TrxR (Trx1-3) are broadly expressed and can be found intra- and extracellularly. The thioredoxin system may effectively regenerate proteins that were inactivated by oxidative stress [74]. Trx acts by reducing ROS as well as controlling apoptosis and inflammatory response. A pivotal role of Trx has been reported in the regulation of endothelial cell survival, protecting the cells from laminar shear stress, and inhibiting the activation of c-Jun N-terminal kinase (JNK) and p38 mitogen-activated protein kinase (p38 MAPK) consequent to tumor necrosis factor alpha (TNF-α) exposure [3]. Trx was also found in medial smooth muscle cells of coronary arteries of healthy humans, while a diffused expression pattern was evident in atherosclerotic arteries [73]. The system was also reported to exert an essential role in regulating metabolic processes, insulin signaling, blood pressure regulation, and inflammation [73].

#### 2.2.5. Peroxiredoxin

Peroxiredoxin (Prx) belongs to a family of cysteine-dependent peroxidases that regulates ROS homeostasis. Prx is capable of reducing peroxides, H_2_O_2_, and peroxynitrite, using electrons provided by thiols such as Trx [79]. Six isoforms have been identified in mammals. Concerning endothelial dysfunction, it has been reported that the silencing of Prx2 in human aortic endothelial cells caused the inactivation of VEGF receptor 2 (VEGFR2) by oxidation, reducing chemotactic mobility and proliferation in response to VEGF [80]. Mice Prx6−/− showed endothelial cell apoptosis and blood vessel damaging after a skin injury, while Prx6 knockdown affected endothelial cell survival after H_2_O_2_ treatment in vitro [81]. It has been also reported that Prx1 showed anti-oxidative and anti-inflammatory properties on bovine aorta endothelial cells exposed to laminar shear stress [82].

### 2.3. Redox-Regulated Transcription Factors

Transcription factors are the core of intracellular signaling, as they integrate multiple inputs from the environment, such as ROS and NO, and translate them into coordinated cellular responses [83]. Among transcription factors, nuclear factor kappa B (NF-κB) or nuclear factor-2 erythroid related factor-2 (Nrf2) have been primarily studied in different experimental models (Stefanie Kohlgrüber Antioxid Redox Signal. 2017). NF-κB is a transcription factor, involved in pro-inflammatory cytokine production and endothelial dysfunction [3,84]. The herbal medicinal product Phytodolor^®^ (STW 1) and its components have shown anti-inflammatory properties on lipopolysaccharide (LPS)-activated human macrophages by inhibiting the translocation of the subunit p65 of NF-κB into the nucleus [85]. IMM-H007 (H007) is a small molecule compound with anti-inflammatory properties regulating endothelium inflammation [86]. It has been reported that H007 significantly reduced monocyte adhesion and transendothelial migration by inhibiting TNFα-mediated inhibitor of nuclear factor kappa B (Iκ-Bα) degradation and NF-κB nuclear translocation, as well as preventing JNK/c-Jun phosphorylation [86].

Bromodomain and extraterminal (BET) proteins are essential for the expression of a subset of NF-kB-induced inflammatory genes. BET mimics, including JQ1+, prevent binding of BETs to acetylated histones and down-regulate the expression of selected genes. JQ1+ decreased NF-kB p65 recruitment to native interleukin-6 (IL-6) and interleukin-8 (IL-8) promoters, proliferation, and migration of primary human pulmonary microvascular endothelial cells [87].

Nrf2 is a redox-regulated transcription factor in the regulation of antioxidant defense systems [84]. Nrf2 interacts with antioxidant response element sequences of genes coding for antioxidant enzymes, including γ-glutamyl cysteine ligase, NAD(P)H quinone oxidoreductase-1, glutathione S-transferase, heme oxygenase-1, uridine diphosphate glucuronosyltransferase, superoxide dismutase, catalase, and glutathione peroxidase-1. These enzymes play a crucial role in protecting the endothelium from ROS induced-endothelial dysfunction [88]. It has been reported that Nrf2-driven free radical detoxification pathways exert an important vasoprotective function against aging, atherosclerosis, hypertension, diabetes, ischemia, and smoking-related cardiovascular diseases [89]. Nrf2 activators can act directly, preventing the interaction of Nrf2 with the Nrf2-binding site of Kelch-ECH-associated protein-1 (KEAP1), like the case of ML334, or they can favor the transcription of Nrf2-targeted antioxidant genes (berberine) or increasing Nrf2 half-life (MG-132) [88]. Pharmacological activation of Nrf2 with sulforaphane (SFN) proved to be effective in endothelium-dependent vasodilation and H_2_O_2_-induced relaxation in vascular beds of aging rats. The pharmacological activation of Nrf2 improved age-related impairment of endothelium-dependent and ROS-induced vasodilation in different rat and human vascular districts by up-regulating Nrf2-related genes and decreasing oxidative stress [90]. Hypoxia-inducible factor-1α (HIF-1α) is a hypoxia-specific transcription factor-induced at the transcriptional, post-transcriptional, and posttranslational levels by hypoxia and can be used to restrict gene expression to ischemic areas [91]. It is implicated in the development of atherosclerosis, acting on endothelial cells (ECs), VSMC, and macrophages [92]. Prolyl hydroxylase (PHD) inhibitors, stabilizers of hypoxia-inducible factors (HIFs), have been used to treat acute organ injuries such as renal ischemia-reperfusion, myocardial infarction, and, in some contexts, chronic kidney disease [93]. Enarodustat (PHD inhibitor) proved to reduce cardiac hypertrophy and fibrosis in a unilateral urinary obstruction (UUO) model in mice [94]. Moreover, enarodustat counteracted kidney fibrosis reducing pro-inflammatory cytokine expression, apoptosis, and improving capillary density in a nephrectomy model [93].

## 3. Oxidative Stress and Cellular Biomarkers for Prevention and Diagnosis of Endothelial Dysfunction

The identification of oxidative stress markers represents a research goal for clinical diagnosis of endothelial dysfunction, prevention, therapy, or monitoring of CVD [50]. Oxidatively modified lipids, proteins, nucleic acids, and activities of antioxidant enzymes are used as biomarkers of oxidative stress [95]. The quantification of ROS is a difficult challenge, given their extremely short half-life. A diffuse method is to measure stable by-products modified under oxidative stress or circulating associated factors [50]. Lipid peroxides, nitrotyrosine, and NO are included among different biomarkers that characterize a dysfunctional endothelium [34].

### 3.1. Lipid Peroxidation

Lipid peroxidation represents a useful marker of oxidative stress because the hydroxyl radical is the most reactive form of ROS oxidizing polyunsaturated fatty acids [95]. Lipid peroxidation can be measured in biological fluids (e.g., blood, serum, plasma, and urine) through the concentration of lipid peroxides themselves or of end products of lipid peroxidation, such as malondialdehyde, isoprostanes, and the malondialdehyde-derived fluorophore (DHP)-lysine [95]. Increased lipid peroxidation is associated with the risk of thrombosis in patients with primary and secondary antiphospholipid syndrome, hypertension-related microvascular changes, and metabolic disease, including obesity, diabetes, and cardiovascular complications [34,96,97,98]. However, depending on the assay used, conflicting results about the usefulness of oxLDL for CVD prediction have been reported in studies on the association of oxLDL with atherosclerosis severity [99].

### 3.2. Peroxynitrite and NO

One of the ROS subsets is represented by reactive nitrogen species (RNS), which includes peroxynitrite (ONOO−), dinitrogen trioxide (N_2_O_3_), and the nitrosonium ion (NO^+^). RNS determine post-translational modifications of proteins and nitrative stress with RNS-induced modifications, such as S-nitrosation and tyrosine nitration, which induce cellular dysfunction [99]. Different nitroproteins have been identified in plasma, but the ELISA for nitrated albumin remains the only clinically validated quantitative assay [100]. NO metabolites, NO synthase inhibitors, and N-acetyl-β-glucosaminidase (NAGase) are biomarkers capable of measuring endothelial dysfunction and oxidative stress in the early stages of impaired response to insulin [101]. NO metabolites and NAGase activity were elevated in glucose intolerance and type-2 diabetes (TD2) patients, while nitrotyrosine is higher only in the TD2 group [101]. Once formed, superoxide anions can inactivate NO, leading to the generation of ONOO- that is a potent oxidant [102]. ONOO- inhibits endothelium-dependent vasorelaxation, reducing the beneficial effects of NO on platelet aggregation and vascular smooth muscle cell proliferation and causing the oxidation of DNA and lipids; altogether, these factors are involved in the development of atherosclerosis [102]. Moreover, it has also been reported that aging increases nitrative stress in resistance arteries, evidenced by elevated ONOO- production in serum of aged mice [103]. ONOO- was responsible for the endothelium-dependent vasorelaxation dysfunction recovered by the treatment with FeTMPyP (ONOO- scavenger) [103].

### 3.3. Glutathione, Glutamyltransferase, Guanine and Low-Density Lipoprotein

In a cohort of 124 healthy nonsmokers, high plasma levels of reduced and oxidized forms of thiols, including glutathione (reduced and oxidized glutathione, GSH and GSSG), cysteine, and the mixed disulfide were found, indicating a significant correlation with endothelium-dependent vasodilation [104]. Another important oxidative stress marker is gamma-glutamyltransferase (GGT). Cellular GGT breaks down extracellular GSH and provides cysteine for GSH de novo synthesis. In this reaction, GGT releases ROS at low levels in the presence of iron or other transition metals [105]. Therefore, an increased GGT activity can represent a marker of antioxidant inadequacy and increased oxidative stress. Scientific evidence suggests that elevated GGT activity is associated with an increased risk of CVD because GGT induces LDL peroxidation [106]. Moreover, the erythrocyte GSH/GSSG ratio is also significantly reduced in the prediabetes condition, indicating increased oxidative stress. In that condition, both urinary 8-hydroxy-2′-deoxyguanosine (8-OHdGuo) and plasma homocysteine are significantly elevated, indicating an association with endothelial dysfunction [107]. The association between urinary oxidized guanine/guanosine and 8-isoprostane levels is also found with myocardial infarction, stroke, and CVD mortality [108]. It has been reported that endothelial dysfunction in pre-eclampsia (PE) is related to the enhanced oxidative stress and oxLDL. Circulating oxLDL and antibodies to oxLDL are associated with atherosclerosis and in PE patients [109]. Oxidized LDL, together with ROS production, is involved in plaque formation and can lead to its instability and, ultimately, to rupture [106]. Finally, high levels of serum GGT have been reported to be associated with cardiovascular risk factors, oxidative stress, immune inflammation, and endothelial dysfunction [105].

### 3.4. Endothelial Dysfunction Markers Associated with Inflammation

Endothelial inflammation is indicated by elevated levels of soluble vascular cell adhesion molecule (sVCAM), soluble intercellular adhesion molecule (sICAM), E-selectin, along with ACE, von Willebrand factor (VWF), a disintegrin and metalloproteinase with thrombospondin motifs 13 (ADAMTS-13), C-reactive protein (CRP), tumor necrosis factor-alpha (TNF-α), and other inflammatory cytokines [34,110,111]. The circulating non-transferrin-bound iron level has been found higher in diabetic patients. Besides, malondialdehyde plasma levels have been reported to be higher in both TD2 and obese groups and to be associated with higher levels of oxidized ascorbate [112]. E-selectin has found elevated in TD2 patients as well as CRP levels, which has been reported to be higher in TD2 and obese patients [112].

### 3.5. Endothelial Progenitor Cells

EPCs can be considered biomarkers per se [34]. During endothelial damage, the number of endothelial progenitor cells increases, whereas endothelial microparticles (EMPs) and circulating endothelial cells (CECs) are reduced [34,113]. EMPs are circulating submicron-sized vesicles secreted by damaged endothelial cells, implicated in vascular inflammation, thrombosis, angiogenesis, and atherosclerosis progression [34,114]. EMPs represent an emerging biomarker of endothelial dysfunction. In hypertensive patients, the high plasma level of EMPs was associated with an impaired glomerular filtration rate [114] and are detected in different circulatory hypoxia-related diseases, including acute coronary syndromes, myocardial infarction and stroke [35].

### 3.6. Endoglin

Endoglin (Eng) is a transmembrane glycoprotein and a co-receptor of transforming growth factor-beta (TGF-β) complex [115]. However, a soluble form of endoglin (sEng) can be produced by the proteolytic action of MMP-14. It has been found on endothelial cells, activated macrophages, activated monocytes, fibroblasts, and smooth muscle cells. It is involved in cardiovascular development, angiogenesis, and vascular remodeling [115]. It has been demonstrated that sEng levels increased both the expression of cell adhesion molecules and the number of rolling leukocytes, and that they impaired endothelial-dependent vascular function [116]. Moreover, sEng levels were higher in blood during endothelial injury or inflammation, but also in other vascular pathological conditions, such as atherosclerosis, hypercholesterolemia, hypertension, and T2D [117]. A clinical study conducted on 288 patients with T2D, hypertension, and healthy controls showed a significant correlation between endoglin and glycemia, glycated hemoglobin, systolic blood pressure, left ventricular hypertrophy and endothelial dysfunction [118]. Transgenic mice with high expression of human sEng showed arterial blood pressure higher than control, but a similar endothelium-dependent vascular function and expression of adhesion molecules [116]. In light of this evidence, further studies are needed to clarify better the role of Eng in the pathogenesis of endothelial dysfunction and CVD.

### 3.7. Uric Acid

Uric acid has been indicated as a well-known oxidative stress biomarker. Uric acid is a breakdown product of purines. The latter are oxidized and catalyzed by the action of xanthine oxidase, in xanthine, and then in uric acid. High concentrations of uric acid may reflect higher levels of xanthine oxidase activity and oxidative stress [119]. The action of xanthine oxidase leads to the generation of superoxide anions and is one of ROS’ principal sources in the human vasculature [119]. Moreover, it has been reported that higher uric acid levels promoted endothelial activation of ECs through the inhibition of eNOS and NO and the induction of NADPH oxidase (NOX) activity [120] that is a complex of seven enzymes, which produces ROS and is localized at the cell membrane [121]. In light of this evidences, uric acid has been proposed as a serum indicator of endothelial dysfunction [122]. Some studies demonstrated that an elevated plasma concentration of uric acid represents a powerful predictor of adverse event rate and mortality in patients with CVD [123,124]. At physiological concentrations, uric acid reduces the oxo-heme oxidant, generated by the peroxide reaction with hemoglobin, protecting erythrocytes from peroxidative damage and lysis [125], and protects against oxidative damage by acting as an electron donor [126]. So, physiological levels of uric acid act as antioxidant, scavenging singlet oxygen and radicals in plasma.

## 4. Epigenetic Regulation in Endothelial Dysfunction and Vascular Damage

The impact of epigenetics in CVD is recently emerging as an essential regulatory role at a different level, from pathophysiology to therapy. Epigenetic studies in CVD were undertaken due to its prominent role in inflammation and vascular involvement and revealed a significant number of modifications affecting the development and progression of CVD [127]. Furthermore, epigenetics contributes to cardiovascular risk factors such as smoking, diabetes, hypertension, and age [127]. Epigenetics refers to all heritable changes in gene regulation without altering DNA sequencing. The primary epigenetic mechanism of human cells includes DNA methylation, posttranslational histone modifications, and non-coding RNA (i.e., long non-coding RNAs and microRNAs) [127]. Growing evidence suggests that endothelial response to blood flow is modulated through epigenetic mechanisms such as histone modification, chromatin remodeling, and microRNAs [128].

### 4.1. DNA Methylation

DNA methylation has been studied extensively and represents a well-understood epigenetic mechanism. During this process, cytosine (C) residues preceding guanosine (G) in the DNA sequence are methylated. CpG-islands are short interspersed DNA sequences with clusters of CG sequences. The abnormal methylation of CpG islands in the promoter region of genes leads to a silencing of genetic information and, finally, to alter cell function [129]. For instance, hyper- and hypomethylation can result in suppression and stimulation of transcription in human endothelial cells, in response to a disturbed flow [128]. As previously described, p66(Shc) upregulation is implicated in hyperglycemia-induced endothelial dysfunction and aging [47,48,49]. Its overexpression was reported to be epigenetically regulated by promoter CpG hypomethylation and nonderepressible, 5-induced, histone three acetylation [48]. Gene silencing of p66(Shc) has been proven to restore endothelium-dependent vasorelaxation, counteracting cell apoptosis by preventing cytochrome c release, caspase 3 activity, and cleavage of poly (ADP-ribose) polymerase in vitro and in vivo, suggesting p66(Shc) as a potential therapeutic target in endothelial dysfunction [48].

### 4.2. Histone Modifications

Histone modifications lead to the modulation of genetic information’s expression through the modification of DNA accessibility [129]. Jumonji domain-containing protein 2A (JMJD2A) catalyzes the demethylation of trimethylated histone H3K9 (H3K9me3) and H3K36 (H3K36me3) and mediates cardiac hypertrophy through the regulation of its target four and a half LIM domains protein 1 (FHL1) [129]. Besides, it has been reported that the epigenetic silencing of the endothelial nitric oxide synthase (eNOS) promotor regulates angiogenesis, through methylation of the lysine residue 27 on histone H3 (H3K27me3). In fact, during hypoxia, eNOS expression increases through the reduction of H3K27me3 by the histone demethylase jumonji domain-containing protein 3 (JMJD3) [129]. Moreover, it has been reported that the silent information regulator 1 (SIRT1) directly binds to the p66Shc promoter (−508 to −250 bp), which represses p66Shc transcription by deacetylating the histone [49]. These findings explain the observed phenomena of reduced expression of SIRT1 and increased expression of p66Shc in many different pathological conditions, including aging, dyslipidemia, and vascular dysfunction [49]. Furthermore, the aberrant histone methylation (H3K4me1, H3K9me2, and H3K9me3) at promoters of Nox4 and eNOS are the leading causes for the persistent up-regulation of these two genes [130].

### 4.3. MicroRNAs (miRNAs)

Recently, the regulatory role of small and long non-coding RNAs has been highlighted. MiRNAs are central orchestrators of these networks [131]. MiRNAs are small non-coding RNAs of approximately 22 bp in length. In general, miRNAs downregulate target gene expression [132]. miRNAs’ biosynthesis is a complex process that starts in the nucleus and is completed in the cytoplasm of the cell [132]. Several miRNAs have been proposed to regulate vascular function and the development of atherosclerosis from different pre-clinical and clinical studies [133].

MiRNAs mediate the adaptation of endothelial cells subjected to shear stress, thereby transforming the mechanical stimuli into intracellular signals [128]. MiRNAs are involved in the regulation of physiological and pathological processes, such as systemic arterial hypertension, atherosclerosis, T2D, and obesity [134]. Experimental evidence showed that dysregulation of microRNAs (miRNAs) has a role in vascular aging [135]. However, the molecular mechanisms of miRNAs are not still well understood [135].

In systemic arterial hypertension (SAH), miRNAs play a significant role in the regulation of key signaling pathways that lead to the hyperactivation of the RAAS, endothelial dysfunction, inflammation, proliferation, and phenotypic changes in smooth muscle cells [134]. Normally, vascular endothelial growth factor (VEGF) is involved in angiogenesis and blood pressure regulation. Some studies demonstrated that VEGF signaling is modified in SAH. Different miRNAs were involved in the regulation of VEGF signaling [136]. In particular, miR-126 is highly expressed in cardiac endothelium. It contributes to angiogenesis and the maintenance of vascular integrity, by targeting the phosphatidylinositol 3′-kinase (PI3K) regulatory subunit p85 β, which leads to modulation of the PI3K/protein kinase B (Akt) signaling pathway in endothelial progenitor cells [137]. Moreover, it has been demonstrated that miR-126 inhibits sprouty related EVH1 domain containing 1 (SPRED-1), another negative regulator of angiogenesis that acts by inhibiting the VEGF signaling pathway [138]. Transgenic mice lacking miR-126 expression showed developmental abnormalities such as vascular leakage, hemorrhaging, and partial embryonic lethality, likely due to miR-126 interaction with pro-angiogenic growth factors [138]. Endothelial cells transfected with a miR-126 inhibitor showed increased inflammation and TNF-α-induced VCAM-1 expression [139]. MiR-15b and miR-16 are also involved in the regulation of angiogenesis and vascular function. Both miRNAs targets VEGF mRNA and are downregulated in hypoxic conditions [140]. Along with VEGF, FGFs are also powerful promoters of angiogenesis [141]. In the plasma of hypertensive patients, up-regulation of miR-505 associates to FGF18 inhibition, thus representing a potential biomarker of pre-hypertension [142]. MiR-17-3p and miR-31 have been reported to be involved in vascular inflammation through the expression of adhesion molecules VCAM-1, ICAM-1, and E-selectin, and the increase of oxidative stress and NO depletion [143,144]. Instead, miR-155 affects endothelium-dependent vasodilation by reducing eNOS expression [145] and adaptive neovascularization [146]. MiR-19a has been reported to display anti-proliferative properties in endothelial cells by inhibiting cyclin D1 mRNA [147]; instead, miR-19b counteracts the TNF-α-induced endothelial cell apoptosis [148]. A possible role in endothelial cell apoptosis has been hypothesized for Let-7g, miR-21, and miR-223 [149,150]. Moreover, in an experimental rat model, a higher level of miR-223 causes hypertension-induced heart failure [151]. Several miRNAs, such as miR-155, miR-146a/b, miR-132/122 cluster, and miR-483-3p, have been demonstrated to be involved in RAAS-mediated cardiovascular inflammation [152]. MiR-145, miR-27a/b, and miR-483-3p inhibit ACE expression [153]. Instead, miR-143/145 clustering is downregulated in the presence of a high level of ACE in SAH [154]. Moreover, miR-483-3p and miR-155 have been reported to be reduced when angiotensin II level is high [155,156]. In SAH, arterial structural remodeling occurs, leading to lumen reduction [157]. The key event in this process is VSMC proliferation and migration following arterial damage [158]. Several miRNAs participate in the regulation of VSMC phenotype. In particular, miR-221 seems to reduce the contractile phenotype of VSMCs in response to platelet-derived growth factor [159]. Some studies reported that higher levels of miR-221 and miR-222 correlated with SAH [160]. Normally, the miR-143/145 cluster is highly expressed in VSMCs and regulates the differentiation of stem/progenitor cells. The downregulation of miR-143/145 cluster, in SAH, may influence the contractile phenotype of VSMCs [161,162]. MiR-133 has been reported to be a negative regulator of proliferation [163] as well as miR-365 that downregulated cyclin D1 expression [164]; instead, miR-21 seems to be a positive regulator of proliferation and survival of VSMCs [165].

A consistent pattern of lower levels of circulating miRNAs was found in heart failure patients with atherosclerotic disease, in particular peripheral arterial disease [166]. In particular, lower plasma levels of miR-18a-5p, miR-27a-3p, miR-199a-3p, miR-223-3p and miR-652-3p have been reported in atherosclerotic patients [166]. Besides, miR-126-5p promotes the replicative regeneration of arterial endothelial cells via suppression of the Notch1 inhibitor delta-like 1 homolog, which prevents atherosclerotic lesion formation and regulates endothelial cell turnover through the inhibition of suppressors of the PI3K pathway [167,168]. Aging is associated with ROS increase, and senescence plays an important role in the pathogenesis of vascular dysfunction [169,170]. Specific changes in the miRNA expression profile of aged mice compared to young ones has been reported [171]. MiR-217 negatively regulates the expression of SIRT1, which is an NAD^+^ dependent deacetylase that regulates gene expression by deacetylation of modified lysine residues on histones, transcription factors, and some transcription cofactors. Some studies show that inhibition of miR-217 in old endothelial cells reduced senescence and increased angiogenesis [172]. Besides, miR-146 negatively regulates NOX4 expression, the main endothelial NADPH oxidase isoform, and its overexpression induces ROS generation, thus favoring senescence [169,173]. Instead, miR-21 upregulation plays a positive role in the regulation of the angiogenic phenotype of microvascular endothelial cells [169,174]. Moreover, miR-125a-5p and miR-125b-5p negatively downregulated endothelin-1 in endothelial cells of hypertensive rats [175]. MiR-155 has been reported to reduce the expression of eNOS and AT1, the two main players in the vascular homeostasis [146]. Other miRNAs, such as miR-10a, -19a, -23b, -17~92, -21, -663, -92a, -143/145, -101, -126, -712, -205, and -155 have been reported to play a critical role in endothelial function and atherosclerosis by targeting genes involved in inflammation, adhesion molecules, cell cycle, proliferation, migration, apoptosis, and NO signaling in endothelial cells [176,177,178,179,180,181].

Endothelial dysfunction and the alteration of the expression of specific miRNAs in endothelial cells also play a pivotal role in the pathophysiology of diabetes. Different miRNAs are involved in the regulation of insulin signaling and blood glucose levels in T2D and participate in lipid metabolism, adipogenesis, and adipocyte differentiation in obesity [182]. Several studies are focusing on the identification of new endothelial dysfunction markers in the blood of diabetic patients. In particular, circulating levels of miR-126 have been negatively associated with diabetes [183]. Furthermore, miR-126, miR-15a, miR-29b, and miR-223 were found specifically downregulated in T2D [184]. Inflammation-related miRNAs are critical because involved in cardiometabolic diseases. It has been reported that the levels of miR-155, miR-10a, and miR-181b were significantly lower in patients with coronary artery disease [185,186,187]. The miR-181b expression is found reduced in endothelial cells from the adipose tissue of a mouse model of obesity, suggesting a positive role in glucose homeostasis and insulin signaling [188]. The restoring of these circulating anti-inflammatory miRNAs counteracts the progression of the disease [189]. Many studies revealed a central role of miRNAs as core regulators of gene expression during cardiac disease [131]. From a clinical perspective, miRNAs may provide valuable diagnostic and prognostic biomarkers [190].

### 4.4. Long Non-Coding RNAs (lncRNAs)

More recently, much interest has been shown on another class of ncRNAs, named long non-coding RNAs (lncRNAs), belonging to a novel heterogeneous class of non-protein-coding transcripts with a length of more than 200 nt and with many different functions. LncRNAs can act as decoys by binding to RNA or proteins, and inhibit or promote transcription through histone and chromatin alteration, alter splicing profiles, or mask miRNA binding sites [191]. LncRNAs play a crucial role in regulating many critical biological processes, such as proliferation, differentiation, migration, and development, thus contributing to the pathogenesis of several human diseases [191]. The association between lncRNAs and cardiovascular disease is just coming to light with reports describing their specific expression in different cardiac diseases. Dysregulation of specific lncRNAs has been shown in both human and rodent models, with encouraging results for vascular disease prognosis and therapy [191]. Some lncRNAs differentially expressed during cardiovascular development also participate in the progression of CVD. Smooth muscle and endothelial cell-enriched migration/differentiation-associated lncRNA (SENCR) and H19 lncRNA, for example, are widely implicated in cardiovascular disease. SENCR has been suggested to affect coronary artery disease (CAD), by playing a role in vascular and endothelial differentiation during development [192]. SENCR was found to be downregulated in VSMCs in a murine model of T2D and its reduced expression associated with premature CAD in humans [193]. H19 is an essential regulator of mammalian development and disease and inhibits cell proliferation [194]. Studies reveal an expression of lncRNA H19 in CVD, although with still unclear mechanisms [195]. The lncRNA HOX antisense intergenic RNA (HOTAIR) facilitates cell proliferation and migration and suppresses apoptosis in endothelial cells [196]. It has been reported to be much lower in endothelial cells from atherosclerotic plaque [196]. Moreover, in atherosclerosis patients, serum level of thymic stromal lymphopoietin was lower and positively correlated with HOTAIR expression in endothelial cells, suggesting a potential therapy for endothelial dysfunction in atherosclerosis [196]. The metastasis-associated lung adenocarcinoma transcript 1 (MALAT1) is a lncRNA highly expressed in different types of endothelial cells and has been implicated in controlling inflammation and angiogenesis. Silencing of MALAT1 reduces endothelial cell proliferation by inhibiting cell cycle progression, decreasing the number of S-phases under basal, hypoxic conditions, and after VEGF stimulation [197]. As interest in the role of lncRNAs increases, the technology aims to enhance their detection, becoming more sophisticated, and the use of RNA sequencing screens has identified promising candidates for therapy [191]. LncRNAs potentially represent a powerful tool for personalized medicine due to their specific expression patterns associated with distinct pathologies [191]. The detection of lncRNAs in circulating exosomes opens interesting perspectives, in terms of signaling regulation and intercellular communication, and for further translational applications to diagnostics. However, several limitations and challenges remain to be resolved before lncRNAs reach a clinical application [191]. Summary of biomarkers in Table 2.

## 5. New Therapeutic Approaches Targeted Endothelial Dysfunction

Several factors appear to affect endothelial dysfunction, and different strategies have been proposed to change these factors and counteract it. Other than conventional drug therapies, lifestyle and dietary interventions are majorly encouraged, especially in age-related vascular endothelial dysfunction [198]. For example, regular exercise and caloric restriction can exert beneficial effects increasing NO bioavailability, eNOS, and SOD activity, as well as reducing oxidative stress, cholesterol, and systolic blood pressure. Besides, several ‘anti-aging’ nutraceutical compounds have been recently proposed to counteract oxidative stress and senescence [40]. For example, both omega 3 polyunsaturated fatty acids (ω-3 PUFA) and extra virgin olive oil (EVOO) have been reported to exert numerous metabolic and cardiovascular benefits in the elderly due to their high content in antioxidant compounds [199,200,201,202]. In old rats, the treatment prevented aging-induced endothelial dysfunction, oxidative stress, inflammation, and vascular insulin resistance through activation of the PI3K/Akt pathway and decreasing the response to the vasoconstrictor angiotensin II [202]. Moreover, the Citrus flavonoid naringenin (NAR) has been demonstrated to exert anti-senescence effects, and, when administered to old mice, it reduced ROS production in myocardial tissue, inflammation, and vascular remodeling [203]. NAR beneficial effects are attributable to its similarity to the natural structure of SIRT1 activator resveratrol, an enzyme involved in many physiological functions and reported to be depleted in endothelial dysfunction [203]. An experimental study on *Moringa oleifera* (MOI) suggested a vascular protective effect of MOI seeds against the aging-related vascular dysfunction through increased Akt signaling, endothelial NO synthase activation and downregulation of arginase-1, improving the endothelial-dependent relaxation of mesenteric arteries [204]. In aged mice, the restoration with the NAD^+^ booster nicotinamide mononucleotide (NMN) of the depleted NAD^+^ levels demonstrated vasoprotective effects [171,205]. NMN increased endothelium-dependent vasodilation and counteracted oxidative stress [171]. Unfortunately, current conventional and antioxidant drugs are not entirely effective in reversing endothelial dysfunction. Therefore, besides standard therapies, different new approaches have recently been proposed in endothelial dysfunction and CVD therapy.

### 5.1. Nanotechnologies for Drug Delivery Systems

Nano therapies based on nanoparticles are very promising for the treatment of cardiovascular diseases. Nano-medicine is an advanced version of a conventional drug that includes the development and application of nanomaterials and nanotechnologies [59]. Nano-medicine is highly specific for targeted drug delivery, improves drug bioavailability, minimizes associated side effects, and reduces costs [59]. A broad spectrum of nanoparticles ranging from liposomes and niosomes to polymers, lipid and organic polymer hybrids and precursors, carbon nanotubes, quantum dots, metal, metal oxides including nanoparticles and biological molecules are used for vascular diseases [206]. An advanced application of nano-medicine may be described by the enhanced therapeutic efficacy of statins against CAD [207]. Broz et al. reported using a vesicle system to deliver high-dose statin, reducing toxicity in other tissues. Vesicles were loaded with pravastatin and surface-functionalized with an oligonucleotide with a high affinity for inflammatory macrophages [208]. Instead, Leuschner et al. demonstrated the efficacy of nanoparticle-assisted systemic delivery of a short interfering RNA (siRNA) silencing C-C chemokine receptor type 2 (CCR2), a chemokine receptor for monocyte recruitment, that significantly decreased plaque burdens [209]. As described above, synthetic siRNA can effectively inhibit its target gene, but it cannot pass through the cell membrane because of its large size and negative charge [210]. Therefore, the delivery of siRNA into cells represents a significant challenge in clinical application, and nanoparticles may provide a solution. The use of liposomes may go over this problem by extending the circulatory half-lives of drugs and improving their vascular endothelium deposition. For example, liposomal glucocorticoid therapy reported by Lobatto et al. demonstrated a significant reduction of inflammation in atherosclerotic plaques [211]. Moreover, the use of light-activated dextran-coated iron-oxide nanoparticles, in an apolipoprotein E (ApoE) knockout mouse model, has been reported to be effective in reducing inflammation through macrophage ablating, without causing significant skin toxicity [212]. Dextran-coated iron-oxide nanoparticles, loaded with phototoxic agents, induced macrophage death within atherosclerotic plaques once irradiated [213]. PARP, PTPase, ROCK, geranylgeranyltransferase, and transketolase are promising targets for the treatment of cardiovascular disorders [51,214,215]. Oxidant-mediated activation of PARP plays a fundamental role in the development of endothelial dysfunction [216]. PARP inhibitors such as PJ-34 and INO 1001 act against diabetes, hyperhomocysteinemia, hypertension, and aging [216,217,218,219]. Human recombinant interleukin-37 (IL-37), an anti-inflammatory cytokine of the interleukin-1 family, has been reported to favor vascular endothelial function in old age by increasing NO bioavailability and the phosphorylation of AMP-activated kinase (AMPK), as well as reducing levels of ROS [220]. Etanercept (TNF-α antagonism) treatment has been demonstrated to be useful to improve aging-impaired endothelium-dependent relaxant responses in rats [221]. Akt activation stimulates eNOS phosphorylation, increases NO production, and reduces oxidative stress [222]. DAQ B1, an activator of Akt, reduces oxidative stress, and prevents hypertension and diabetes [223,224]. BMOV is an inhibitor of PTPase that activates eNOS, opening ATP-sensitive channels, and consequently decreasing oxidative stress [225,226]. ROCK inhibitors enhance wound healing of the corneal endothelium [53], and clinical trials with cell-based treatment for corneal endothelial dysfunction are ongoing [227,228]. GGTI-298 is an inhibitor of geranylgeranyltransferase-I, it counteracts the activation of some GTPases of the Rho family such as Rho A and Rac1, which cause eNOS inactivation and therefore NO depletion with decreased ROS release [229]. Benfotiamine is a transketolase activator that prevents the vascular accumulation of advanced glycation end-products (AGE) and the induction of pro-apoptotic caspase-3 [230,231].

### 5.2. Mitochondrial and Oxidative Stress-Targeted Therapies

#### 5.2.1. Antioxidant Therapies

Due to the lack of improvement in cardiovascular outcomes by using ‘traditional’ exogenous antioxidants, such as vitamin C and E, to sustain antioxidant activity in CVD, several studies have shifted their attention on the development of new compounds that target antioxidants or mimic endogenous antioxidant enzymes to counteract oxidative stress [232].

##### Mitochondrial Targets

Among these new therapeutic approaches for endothelial dysfunction is that of delivering antioxidants (as ubiquinol or α-tocopherol) to the mitochondria [88] with lipophilic cations such as mitoquinone (MitoQ) or MitoE2 [233,234]. MitoQ has been demonstrated to reverse in vivo aortic stiffness in old mice, likely counteracting the age-related aortic elastin degradation suggesting that mitochondria-targeted antioxidants may represent a novel, promising therapeutic strategy for decreasing aortic stiffness with primary aging and age-related clinical disorders in humans [235].

Cardiolipin has been proposed as a target of possible pharmacological approaches in CVD [236,237,238,239,240,241,242,243,244,245,246]. Elamipretide (ELAM) (also referred to as Bendavia or SS-31) is a tetrapeptide, soluble in water [247], which can localize itself selectively in the inner mitochondrial membrane binding cardiolipin and stabilizing it, thus preventing its dissociation from electron transport chain [248]. ELAM, administered subcutaneously, has proven its efficacy in several animal models. ELAM shows efficacy in reducing infarct size of approximately 50% in a rabbit model when administered 20 min before reperfusion [61]. In a model of porcine metabolic syndrome, ELAM can protect coronary endothelial cells from apoptosis and restore subendothelial vascularization, although it can only partially restore myocardial perfusion [249]. In a dog model of heart failure, ELAM improves left ventricle function normalizing heart failure plasmatic markers [250]. ELAM has been used in a swine model of atherosclerotic renovascular hypertension (ARVH) normalizing medullary and cortical oxygenation, reducing fibrosis, and raising microvascular perfusion [251]. In the same model, a reduction of myocardial hypertrophy and fibrosis is also documented, which improved left ventricle relaxation and filling capacity during the diastolic phase [246]. In a rat renal ischemia/reperfusion model, ELAM protected tubular cells from oxidative damage accelerating post-reperfusion recovery [245]. Protection of mitochondria by ELAM restores cardiolipin content in renal endothelial cells by increasing their number, reducing vessel loss, and ameliorating renal perfusion in a porcine metabolic syndrome model [252]. Despite these pre-clinical promising results, clinical studies on humans reported conflicting results. In the EMBRACE ST-elevation myocardial infarction (STEMI) study, a double-blind phase 2a trial testing infusion vs. placebo in patients undergoing primary percutaneous coronary intervention, ELAM failed to reduce the size of myocardial infarct [253]. In phase I double-blind placebo-controlled trial, ELAM was tested in patients with heart failure with a reduced ejection fraction, and ELAM plasma concentrations correlated favorably with changes in left ventricular volume [254]. The PROGRESS-HF Phase 2 Trial was a double-blind placebo randomized trial that tested different doses of ELAM (4 mg and 40 mg) in 71 patients with heart failure (HF), with reduced ejection fraction (HFrEF). The primary outcome of the study was the change in Left Ventricular End Systolic Volume (LVES) at four weeks. Although the drug was well tolerated, neither 4 mg nor 40 mg of ELAM significantly improved the LVES, compared to the placebo [255]. Two other European studies evaluated ELAM in patients with congestive HF. Although they are completed, their results have not been published yet (www.clinicaltrial.gov, NCT02814097, NCT02914665). Several other trials on ELAM and have been completed, and their results are yet to be published (www.clinicaltrial.gov, NCT02788747, NCT02814097, NCT02914665).

##### Antioxidant Enzyme Mimics

Tempol, an SOD mimetic, has proven effective in diabetes-associated microvascular complications and in restoring endothelial vasorelaxation in alloxan-induced diabetic rabbits [232]. Ebselen, a lipid-soluble seleno-organic compound that mimics the activity of GPx1, has reported being effective in counteracting atherosclerosis of diabetic ApoE KO mice [256]. Ebselen analog, diphenyl diselenide, has been reported to be effective against hypercholesterolemic LDL receptor KO mice, reducing atherosclerotic lesions, oxidative stress, and inflammation and favoring vascular function [257].

The same promising results are reported for the use of Trx mimetics. The administration of recombinant human Trx (rhTRX) has proven effective in counteracting hypertension in aged wild-type mice [258]. It has been reported that the supplementation of Trx using thioredoxin mimetic peptides (TMP) counteracted ROS production and restored the VEGF-A-induced migration, proliferation, survival of hyperglycemic ECs [259].

As previously described, the pivotal role of p66Shc in mitochondria dysfunction and the inverse relationship between p66Shc and SIRT1 activity, which in turn reduces oxidative stress and endothelial dysfunction [260], have suggested the use of SIRT mimetics as a strategy to counteract endothelial dysfunction and CVD progression [88].

##### VEGF-VEGFR2 Signaling Targets

Vascular endothelial growth factor (VEGF) can promote endothelial cell proliferation, vascular permeability, and regulate thrombus formation. Past pre-clinical studies demonstrated that serum VEGF levels were significantly elevated in coronary artery disease and atherosclerosis [261]. High VEGF levels can cause the growth of atherosclerotic lesions and plaque rupture in animal models [261]. VEGF receptor 2 (*VEGFR2*) is the principal receptor of VEGF in blood vessels. VEGF-VEGFR2 signaling requires vascular development and homeostasis [261]. The other receptor VEGFR1 exerts antiangiogenic activity and is required for proper vasculature development [262].

The existence of an association between specific VEGFR2 polymorphisms and atherosclerotic cardiovascular diseases in humans has recently been reported [261]. Decreased levels of VEGFR2 in ox-LDL-treated ECs suggest the involvement of VEGFR2 in adaptation to oxidative stress [263]. Some studies have documented that cigarette smoke (CS)-induced oxidative stress and inflammatory responses are increased by inhibition of VEGFR2, thanks to the interaction between CS-derived ROS/nitrogen species and VEGFR2 causing posttranslational modifications of VEGFR2 and blocking of downstream signals, ultimately determining endothelial dysfunction [264]. Moreover, it has been reported that the inhibition of VEGFR-2 by a specific kinase inhibitor (NVP-AAD777) increased the CS-induced oxidative stress and inflammation in mouse lung by reducing eNOS activity and impairing VEGF-induced endothelial cell migration and angiogenesis [265]. It is well known that ROS generated from hyperglycemia promotes ligand-independent phosphorylation of VEGFR2, causing the impairment of the responses of endothelial cells to exogenous VEGF, as demonstrated in a mouse model of diabetes [266]. The treatment with the antioxidant N-acetyl-L-cysteine (NAC) can revert the impairment and restore VEGFR2 signaling, evidencing the implication of oxidative stress in this pathological process [266]. It has also been reported that homocysteine (Hcy)-induced endothelial cell dysfunction was counteracted by astaxanthin (ATX) inducing the activation of VEGF-VEGFR2-FAK signaling axis in human ECs [267].

### 5.3. NOX-Targeted Therapies

NADPH oxidase can also be a promising target for oxidative stress-induced endothelial dysfunction. Triazolopyrimidines are selective inhibitors of NADPH oxidase activity [88]. They inhibit NADPH oxidase-derived ROS in vitro and counteract atherosclerosis [268]. It has been reported that the pan-NOX inhibitors VAS compounds (VAS2870 and its analog VAS3947) demonstrated a strong antiplatelet effect, inhibiting platelet aggregation by blocking PKC downstream signaling. The same finding was also reported in an animal model in which VAS compounds prevented thrombus formation [269]. Moreover, VAS2870 has been reported to be effective in counteracting amylin-induced reduction of endothelial responses in rat mesenteric arteries [270]. A novel pan-NOX-inhibitor, APX-115, has proven to have significantly improved insulin resistance in diabetic mice, similarly to how GKT137831 positively affected oxidative stress [271]. GKT137831, an inhibitor of NOX1 and NOX4, is under clinical trial and has been reported to reduce oxidative stress and diabetic vasculopathy [272]. GKT137831 has been reported to protect lung tissue damage after ischemia and reperfusion injury in a murine model [273]. Nox2 inhibitors (CPP11G and CPP11H) has proven to counteract ROS production by inhibiting stress-responsive MAPK signaling and downstream AP-1 and NF-κB nuclear translocation in human ECs. Nox2 inhibition also improved hind-limb blood flow in mice, reducing ROS level and inflammation after TNF-α administration [274]. Besides, NOX-2 inhibitor gp91ds-tat, combined with the ROS scavenger N-acetyl-cysteine (NAC), has proven to be effective against platelet activation induced by prolonged exposure to oxLDL-associated oxidized phospholipids [275]. GLX351322, a selective inhibitor of NOX4, has been suggested to be effective in T2D [276], while Plumbagin, another Nox4 specific inhibitor, has been reported to counteract oxidative stress-induced endothelial dysfunction and preadipocyte apoptosis under hyperinsulinemic conditions [277,278]. Moreover, rutin, a glycoside of quercetin, protected from endothelial dysfunction through inhibition of NOX4 [279]. These inhibitors are able, in turn, to counteract mitochondrial dysfunction and atherogenesis [280,281].

### 5.4. Gene Targeted Therapies

As described above, several miRNAs are involved in vascular diseases as they regulate vascular cell differentiation, migration, proliferation, and apoptosis through their target genes [282]. Based on this evidence, miRNAs could either be possible biomarkers for diagnosis or therapeutic targets in endothelial dysfunction [283]. Difficulties concerning the clinical use of miRNAs derive from their multiple target genes, making miRNA-based therapy very difficult [284]. For this reason, it is crucial to understand their specific gene targets and signaling pathways to make possible, effective therapeutic strategies based on miRNAs [285]. The major strategies for miRNA-based therapies are focused either on the restoration of suppressed genes by reducing or inhibiting specific miRNAs, or on the suppression of target genes responsible for the pathological condition [286].

The most common strategy to inhibit miRNAs is the use of an oligonucleotide that is complementary to the miRNA target (i.e., anti-miRNAs) [287,288,289]. Other studies show the possibility to use miRNA mimics as sort of synthetic, non-natural nucleic acids that bind the unique sequence of the mRNA target in a gene-specific manner, in order to downregulate gene expression; still not tested in vivo [290]. Chemical modification of miRNA inhibitors is crucial for their application in vivo. There are three ways to chemically modify miRNAs to enable their inhibitive function in vivo [133]. The first class of anti-miRNAs is conjugated with cholesterol (antago-miRNA) to facilitate cellular uptake [291]. The other classes entail the use of oligonucleotides with locked nucleotides acid (anti-miRNAs) or 2′-O-methoxyethyl phosphorothioate modifications [287,288,289]. Until today, more than 800 human miRNAs have been identified [133]. There are still no clinical studies using miRNAs for therapeutic purposes in CVD, but there is positive evidence of their use in animal models. In particular, the administration of an adeno-associated virus expressing miR-1 in rats with cardiac hypertrophy induces the regression of hypertrophy, compared to a control group; several weeks after treatment, the reduction of myocardial fibrosis, an improvement in calcium handling, inhibition of apoptosis, and inactivation of the mitogen-activated protein kinase signaling pathways are observed [292].

Interestingly, the clinical use of high miRNA-101 expression ameliorates fibrosis and cardiac function in post-infarcted rats [293].

Moreover, the inhibition of miR-652, by silencing, protects the heart against pathological remodeling and improves heart function in a mouse model with established pathological hypertrophy and cardiac dysfunction [294]. Finally, another study demonstrated that administration of the inhibitors of miR-29a or -29c (antagomirs) together with drug treatment Pioglitazone, a peroxisome proliferator-activated receptor (PPAR)-gamma agonist, significantly reduced myocardial infarct size and apoptosis [295]. Adeno-associated viral vectors for targeting miRNAs in pre-clinical models demonstrated promising results, but their use to deliver lncRNAs remains to be determined [191].

During the last few years, significant clinical and conceptual progress has been made in cardiovascular gene therapy [60].

The first gene-drug approved was Glybera, which was used to treat severe lipoprotein lipase deficiency, and it represented one of the milestones for the entire field of gene therapy [296]. A new generation of cardiovascular clinical trials is primed to evaluate the potential gene target for CVD, such as atherosclerosis, ischemia, and heart failure [60].

The most promising candidates for therapeutic vascular growth seem to be members of the VEGF and FGF families, hepatocyte growth factor (HGF), and gene therapy approaches combined with cell therapy [60]. Moreover, several randomized controlled trials have tested either naked plasmids or adenoviral vectors to deliver VEGF for the treatment of severe coronary heart diseases, such as EuroinjectOne, KAT, REVASC, NOTHERN, NOVA, and VEGF-Neupogen [297,298,299,300,301,302]. Currently, there are some angiogenic gene therapy trials (KAT301, ASPIRE, Neovasculgen, HGF-X7, JVS-100) that are either ongoing or have recently reported results [303,304,305,306,307,308].

Several trials are currently recruiting patients for gene therapies targeting the cardiovascular system. The targets thus far include SERCA2a, Adenylyl cyclase class-type 6, and VEGF [91]. The very first clinical trial of a gene therapy targeting SERCA2a occurred in 2007 and involved nine patients with advanced heart failure receiving a lone intracoronary infusion of Adeno-Associated Virus Serotype 1 (AAV1)/SERCA2a. It was a multicenter trial designed to evaluate the biological effects and safety profile of the gene transfer. This study found a quantitative biological benefit and an acceptable safety profile. The phase 1/2 study was shown to significantly improve left ventricular remodeling in 39 patients suffering from severe heart failure (NCT00454818). The Phase 2b of this study, called “Calcium Upregulation by Percutaneous Administration of Gene Therapy in Cardiac Disease 2” (CUPID 2), randomly assigned 250 patients to receive either placebo or 1 (AAV1)/SERCA2a [309]. CUPID 2 is one of the largest trials about gene transfer, and, despite the promising results of the earlier phases, it failed to prove a benefit in patients with heart failure and reduced ejection fraction, treated with 1 (AAV1)/SERCA2a. Gene therapy provides elevated and sustained production of the proteins in the target area [91].

Nonetheless, researchers should be cautious when working with angiogenic factors to avoid the formation of leaky or aberrant blood vessels [91]. Gene constructs have been developed so that they can turn gene expression on or off depending on the cellular environment, in order to overcome this limitation. An example is gene activation that responds to hypoxia because of ischemia. As previously described, HIF-1α is a hypoxia-specific transcription factor that is induced by hypoxia and can be used to restrict gene expression to ischemic areas. As blood flow and oxygenation are restored to the hypoxic area, transgene expression would gradually decrease, limiting the chance of undesirable blood vessel formation in areas that do not require angiogenesis [91]. A summary of new strategies in the clinical treatment of endothelial dysfunction is shown in Table 3.

## 6. Future Perspectives and Conclusions

Endothelial dysfunction is a primary step during atherogenesis, and defects in vascular integrity and homeostasis induce the progression of vascular diseases. It is becoming increasingly clear that the discovering of novel targets or specific and safe biomarkers represents a potential tool in cardiovascular research. Technological advances are promising, especially nanotechnology and gene-targeted therapies, and several attempts have been made to select appropriate targets and to design specific tools to counteract endothelial dysfunction. Several clinical trials to test the different pharmacological approaches described above, in various pathological conditions, are still ongoing. Although NOX inhibitors did not provide satisfying results in some previous clinical studies, some phase I trials are still in progress for the GKT137831 (NCT04327089). The early intracoronary administration of the ROCK inhibitor Fasudil is under investigation during primary percutaneous coronary intervention in patients with ST-segment-Elevation Myocardial Infarction (NCT03753269). Numerous randomized studies in progress evaluate the effects of dietary supplementation with MitoQ in several conditions, including diastolic dysfunction, peripheral arterial disease, hypertension, chronic kidney disease (CKD), heart failure with preserved ejection fraction (NCT03586414, NCT03506633, NCT04334135, NCT02364648, NCT03960073). A phase 2 randomized trial examines the supplementation with nicotinamide riboside, which has SIRT mimetic effects, for the treatment of arterial stiffness and elevated systolic blood pressure in patients with moderate to severe CKD (NCT04040959). One multicentric European phase 2 randomized trial is trying to assess the efficacy and safety of catheter mediated, adenovirus-mediated, endocardial gene transfer of the vascular endothelial growth factor-D (AdVEGF-D), to treat patients with refractory angina, to whom revascularization cannot be performed (NCT03039751). The EXACT trial is an American multicentric open-label phase 1/2 trial that aims to treat refractory angina with the direct epicardial delivery to the ischemic myocardium of AdVEGF-All6A+. This replication-deficient adenovirus vector expresses a cDNA/genomic hybrid of human VEGF (NCT04125732). Even though the outlook on future therapeutic strategies seems promising, many challenges remain to overcome before these new technologies can be used in everyday clinical practice. 

## Figures and Tables

**Table 1:** Oxidative stress mechanisms in cardiovascular diseases.

Pathological Condition	Relevant Oxidative Stress Mechanisms	References
**Obesity**	Reactive oxygen species (ROS)-induced downregulation of Nitric Oxide (NO) activity.	[10]
	Perivascular adipose tissue is responsible for tumor necrosis factor alpha (TNF-α) and interleukin (IL)-6 secretion, which sustain a low grade inflammatory state and an increased ROS production.	[11,12]
	In morbidly obese patients, arginase is up-regulated. Increased arginase activity competes with endothelial NO synthase (eNOS), whose main substrate for NO production is arginine; thus, reducing NO availability.	[13,14]
**Diabetes and hyperglycemic conditions**	Hyperglycemia is involved in mitochondrial generation of superoxide anion (O^−.^) which contributes to diabetic endothelial dysfunction through four main pathways: the polyol pathway, increase in intracellular production of advanced glycation end products (AGEs), activation of the protein-kinase C (PKC) pathway, the hexosamine pathway	[15,16]
	Hyperglycemia determines an increased ROS production that leads to mitochondrial DNA damage in endothelial cells. A so-determined increase in mitochondrial fission impairs electron-transport chain, which causes an altered nicotinamide adenine dinucleotide phosphate (NADH)\ flavin adenine dinucleotide (FADH_2_) ratio	[17]
	Hyperglycemia triggers a chronic vascular inflammatory state sustained by TNF-α, interleukin-1 beta (IL-1β), interleukin-6 (IL-6), cluster of differentiation 36 (CD36), monocyte chemoattractant protein-1 (MCP-1), and mediated through the up-regulation of the nuclear factor kappa-light-chain-enhancer of activated B cells (NF-κB) pathway, which leads to endothelial cell apoptosis.	[18,19]
	In the pathogenesis of diabetes, an altered expression of several microRNAs has been demonstrated. A reduced lethal-7 (let-7) microRNA (miRNA) and miR-126 expression and an increased miR-200 expression have been linked to β-cells impairment, insulin resistance, chronic inflammation, and vascular oxidative stress.	[20,21,22,23]
	Insulin resistance is generated by ROS mediated activation of several pathways, such as p38 mitogen-activated protein kinase (p38 MAPK), extracellular signal-regulated kinase (ERK), IkB kinase (IKK). These pathways converge on phosphorylation of insulin receptor substrate proteins, which determine alterations in insulin signal transmission.	[24,25]
	High insulin levels upregulate the MAPK pathway, which is involved in plaque formation and vascular smooth muscle cells (VSMC) hypertrophy and proliferation.	[26]
**Dysmetabolic conditions**	ROS have been associated to low-density lipoproteins (LDL) oxidation, generating oxidized low-density lipoproteins (ox-LDL), which are removed from the systemic circulation by binding to scavenger receptor on macrophage surface. This elicits inflammatory response through NF-κB pathway and determines formation of foam cells.	[27,28]
	Ox-LDLs drive caspase activation and endothelial cell apoptosis.	[29]
	Hyperuricemia contributes to endothelial dysfunction mainly through NO depletion. The reaction between uric acid (UA) and NO generates 6-aminouracil, thus, reducing NO availability. UA is also responsible for increased arginine degradation which is the main eNOS substrate for NO synthesis.	[30]
**Cigarette Smoke**	Cigarette smoke (CS) is a suppressor of endothelial NO synthase eNOS and determines reduction in NO bioavailability. It is also responsible for ROS production, tissue remodeling, and increased expression of adhesion molecules and prothrombotic factors.	[31]
**Hypertension**	Chronic inflammation produces inflammatory chemokines, such as IL-6, IL-1β, TNF-α, and interleukin-17 (IL-17), which promote oxidative stress and recruit macrophages, T and B lymphocytes that are responsible for ROS production and vascular fibrotic remodeling.	[32,33]
	Damage to the endothelial cells that release increased levels of endothelial microparticles (EMP), which causes an impaired glomerular filtration rate and is implicated in vascular inflammation, thrombosis, angiogenesis, and atherosclerosis progression.	[34,35]
	Hypertensive inflammation induces NADPH oxidases (Nox), one of the most important sources of superoxide anion (O^−^) in endothelial dysfunction. Superoxide anion react with NO forming peroxynitrite, which oxidizes the 4-tetrahydrobiopterin (BH_4_), a cofactor of eNOS; thus, determining the uncoupling of eNOS and a decreased NO bioavailability.	[36,37]
	NO is able to modulate response to angiotensin II and to invert angiotensin II-induced arteriolar contraction.	[38]
	Aldosterone has proinflammatory effects that are mediated through the mineralocorticoid receptor (MR). Aberrant activation of MR mediates endothelial and organ damage, directly, or through angiotensin 1.	[39,40]
Aging	Endothelium-dependent dilation (EDD) is decreased with aging because of a reduced NO bioavailability. NO production in older adults are reduced under baseline resting conditions.	[41]
	Aging increases the production of reactive oxygen species in the face of unchanged or reduced antioxidant defenses.	[41]
	Age-related endothelial redox changes affect NF-kB, whose activation induces transcription of pro-inflammatory cytokines that can further suppress endothelial function, thus, creating a vicious feed-forward cycle.	[42]
	Genomic instability, telomere dysfunction or DNA damage has been shown to trigger cell senescence via the p53/p21 pathway and result in increased inflammatory signaling in arteries from older adults.	[42]

**Table 2:** Biomarkers of endothelial dysfunction.

Type	Function	References
Lipid peroxidation	indicates reactive oxygen species (ROS) overproduction	[95]
NO	depleted in endothelial dysfunction	[101]
Glutathione	the reduced GSH/GSSG indicates oxidative stress	[104]
oxGua	indicates ROS overproduction	[108]
8-isoprostane	indicates ROS overproduction	
oxLDL	indicates ROS overproduction	[109]
EPCs	increased in endothelial damage	[34]
EMPs	depleted in vascular inflammation, thrombosis, angiogenesis and atherosclerosis progression	[34,114]
CECs		[113]
Endoglin	secreted in endothelial injury and inflammation process	[117]
Uric acid	indicates oxidative stress	[119]
GGT	increased in oxidative stress	[105]
JMJD2A	mediator of cardiac hypertrophy	[129]
jmjd3	mediator of angiogenesis	
miR-126	increased in angiogenesis and regulates vascular integrity	[137]
miR-15b	negative regulators of angiogenesis and vascular function	[140]
miR-16		
miR-505	increased in hypertension	[142]
miR-17-3p	increased in vascular inflammation	[76,77]
miR-31		[77]
miR-155	negative regulator of endothelium-dependent vasodilation	[143,146]
miR-19a	antiproliferative activity in ECs	[147]
miR-19b	counteracts EC apoptosis	[148]
Let-7g	possible role in EC apoptosis	[149,150]
miR-21	possible role in EC apoptosis and positive regulator of VSMC proliferation and survival	[149,150,165]
miR-223	possible role in EC apoptosis	[149,150,151]
miR-155	involved in RAAS-mediated cardiovascular inflammation	[152,153,154,155,156]
miR-146a/b		
miR-132/122		
miR-483-3p		
miR-145	inhibit ACE expression	[153,155]
miR-27a/b		
miR-143/145	negative regulator of ACE expression and of VSMC contractile phenotype	[154,161,162]
miR-221	reduces VSMC contractile phenotype	[159]
miR-222	increased in hypertension	[160]
miR-133	negative regulator of VSMC proliferation	[163]
miR-365	downregulates cyclin D1	[164]
miR-18a-5p	decreased in atherosclerotic disease	[166]
miR-27a-3p		
miR-199a-3p		
miR-223-3p		
miR-652-3p		
miR-126-5p	promotes EC regeneration and turnover	[167,168]
miR-217	negative regulator of SIRT1 expression	[172]
miR-10a	target genes involved in EC inflammation, adhesion molecules, cell cycle, proliferation, migration, apoptosis, and nitric oxide signaling	[176,177,178,179,180,181]
miR-23b		[178,180]
miR-17~92		[177,180,181]
miR-663		[180,181]
miR-92a		[177,179,181]
miR-101		[180,181]
miR-712		[108,113]
miR-205		[177,181]
miR-155		[180,181]
miR-15a	downregulated in T2D	[184]
miR-29b		
miR-181b	decreased in patients with CAD and positive role in glucose homeostasis and insulin signaling	[187,188]
SENCR	regulates VSMC and EC differentiation, downregulated in T2D and CAD	[192,193]
H19	increased in CVD	[125,126]
HOTAIR	reduced in ECs from atherosclerotic plaque	[196]
MALAT1	induces EC proliferation and angiogenesis	[197]

Abbreviations: ROS, reactive oxygen species; GSH/GSSG, reduced/oxidized glutathione; oxLDL, oxidized low-density lipoproteins; EPCs, endothelial progenitor cells; EMPs, endothelial microparticles; CECs, circulating endothelial cells; GGT, gamma-glutamil transferase; JMJD2, lysine-specific demethylase 4A; jmjd3, Jumonji domain-containing protein D3; SIRT1, sirtuin 1; T2D, type 2 diabetes; CAD, coronary artery disease; SENCR, smooth muscle and endothelial cell enriched migration/differentiation-associated LncRNA; CVD, cardiovascular diseases; H19, H19 long noncoding RNA; HOTAIR, HOX long noncoding RNA; MALAT1, Metastasis Associated Lung Adenocarcinoma Transcript 1 long noncoding RNA.

**Table 3:** New strategies in the clinical treatment of endothelial dysfunction.

Class	Type	Mechanism of Action/Results	Target	References
**Nano-medicine**	Liposomes Niosomes Polymers Carbon nanotubes	drug carrier and delivery	drugs	[59,206,207]
**PARP inhibitors**	PJ-34	counteracts reactive oxygen/nitrogen species	poly (ADP-ribose) polymerase	[218]
	INO 1001	against T2D, hyperhomocysteinemia, hypertension	poly (ADP-ribose) polymerase	[219]
	DAQ B1	reduces oxidative stress and prevents hypertension and T2D	Akt	[223,224]
**PTPase inhibitors**	BMOV	regulates opening ATP-sensitive channels decreasing oxidative stress	eNOS	[226]
**ROCK inhibitors**	Y-27632	counteracts corneal endothelial dysfunction	Rho-associated protein kinase	[227,228]
**Geranylgeranyl transferase inhibitors**	GGTI-298	counteracts eNOS inactivation	Rho A and Rac1	[229]
**Transketolase activator**	Benfotiamine	prevents vascular AGE accumulation and induction of pro-apoptotic caspace-3	AGE	[231]
**Mitochondria-targeted antioxidants**	MitoQ	upregulates mitochondrial dynamics proteins Mfn2 and Drp-1 exerting a protective effect	Ubiquinol α-tocopherol	[234]
	SIRT mimetics	reduction of p66^Shc^ fundamental in the homeostasis of mitochondria	p66^Shc^	[88]
	ELAM	stabilizes the cardiolipin	cardiolipin	[253,254,255]
**NADPH inhibitors**	GKT137831	reduces oxidative stress and diabetic vasculopathy	NOX1, NOX4	[272]
	GLX351322	reduces oxidative stress and T2D	NOX4	[276]
**Gene-target therapies**	EuroinjectOne	fails to improve perfusion in CAD	VEGF-A_165_	[297]
	KAT	improves perfusion in CAD	VEGF-A_165_	[298]
	REVASC	improves perfusion in CAD	VEGF-A_121_	[299]
	NOTHERN	fails to improve perfusion in CAD	VEGF-A_165_	[300]
	NOVA	fails to improve perfusion in CAD	VEGF-A_121_	[301]
	VEGF-Neupogen	fails to improve perfusion in CAD	VEGF-A_165_	[302]
	KAT301	improves perfusion in CAD	VEGF-D	[303]
	ASPIRE	N/A (CAD)	FGF4	[304]
	Neovasculgen	improves perfusion in PAD	VEGF-A_165_	[305]
	HGF-X7	N/A (CAD)	HGF	[306]
	JVS-100	N/A (PAD)	SDF-1	[307]
	SERCA2a	improves left ventricular remodeling in severe heart failure	calcium ATPases	[308,309]

Abbreviations: PARP, poly ADP ribose polymerase; Akt, Protein kinase B; PTPase, protein tyrosine phosphatase; ATP, adenosine triphosphate; ROCK, rho kinase; eNOS, endothelial nitric oxide synthase; NOX, NADPH oxidase; CAD, coronary artery disease; VEGF, vascular endothelial growth factor; FGF, fibroblast growth factor; PAD, peripheral artery disease; HGF, hepatocyte growth factor; SDF-1, stromal cell-derived factor 1.

## Abbreviations

GSH	glutathione reduced
GSSG	glutathione disulfide oxidized
EPCs	endothelial progenitor cells
EMPs	endothelial microparticles
CECs	circulating endothelial cells
EC	endothelial cell
VSMC	vascular smooth muscle cell
T2D	type 2 diabetes mellitus
CAD	coronary artery diseases
CVD	cardiovascular diseases
